# Amoebae as training grounds for microbial pathogens

**DOI:** 10.1128/mbio.00827-24

**Published:** 2024-07-08

**Authors:** Christopher T. D. Price, Hannah E. Hanford, Tasneem Al-Quadan, Marina Santic, Cheon J. Shin, Manal S. J. Da'as, Yousef Abu Kwaik

**Affiliations:** 1 Department of Microbiology and Immunology, University of Louisville, Louisville, Kentucky, USA; 2 University of Rijeka, Rijeka, Croatia; 3 Center for Predictive Medicine, College of Medicine, University of Louisville, Louisville, Kentucky, USA; Instituto Carlos Chagas, Curitiba, Brazil

**Keywords:** *Legionella*, *Coxiella*, *Chlamydia*, *Mycobacterium*, *Rickettssia*, *Salmonella*, *Francisella*, protozoa, *Bartonella*, *Cryptococcus*, *Aspergillus*, *Vibrio*

## Abstract

Grazing of amoebae on microorganisms represents one of the oldest predator-prey dynamic relationships in nature. It represents a genetic “melting pot” for an ancient and continuous multi-directional inter- and intra-kingdom horizontal gene transfer between amoebae and its preys, intracellular microbial residents, endosymbionts, and giant viruses, which has shaped the evolution, selection, and adaptation of microbes that evade degradation by predatory amoeba. Unicellular phagocytic amoebae are thought to be the ancient ancestors of macrophages with highly conserved eukaryotic processes. Selection and evolution of microbes within amoeba through their evolution to target highly conserved eukaryotic processes have facilitated the expansion of their host range to mammals, causing various infectious diseases. *Legionella* and environmental *Chlamydia* harbor an immense number of eukaryotic-like proteins that are involved in ubiquitin-related processes or are tandem repeats-containing proteins involved in protein-protein and protein-chromatin interactions. Some of these eukaryotic-like proteins exhibit novel domain architecture and novel enzymatic functions absent in mammalian cells, such as ubiquitin ligases, likely acquired from amoebae. Mammalian cells and amoebae may respond similarly to microbial factors that target highly conserved eukaryotic processes, but mammalian cells may undergo an accidental response to amoeba-adapted microbial factors. We discuss specific examples of microbes that have evolved to evade amoeba predation, including the bacterial pathogens— *Legionella*, *Chlamydia*, *Coxiella, Rickettssia, Francisella, Mycobacteria*, *Salmonella, Bartonella*, *Rhodococcus*, *Pseudomonas, Vibrio*, *Helicobacter*, *Campylobacter*, and *Aliarcobacter*. We also discuss the fungi *Cryptococcus,* and *Asperigillus*, as well as amoebae mimiviruses/giant viruses. We propose that amoeba-microbe interactions will continue to be a major “training ground” for the evolution, selection, adaptation, and emergence of microbial pathogens equipped with unique pathogenic tools to infect mammalian hosts. However, our progress will continue to be highly dependent on additional genomic, biochemical, and cellular data of unicellular eukaryotes.

## INTRODUCTION

Prokaryotes-protists interactions were initiated at least 2 billion years ago and are ubiquitous in the environment (1, 2). Free-living amoebae prey upon the microbial community as a source of food, which can be scarce in the environment (3–6). However, many microbial pathogens of humans have evolved to avoid predation by amoebae and exploit them as environmental hosts (5, 7–10). In addition to being a rich source of nutrients, microbial residence within amoebae provides a shelter for protection from harmful agents in the environment (10–14). Free-living phagocytic amoebae are thought to be the evolutionary ancestors of macrophages with highly conserved eukaryotic processes (15, 16). Microbial residence within amoebae represents a unique microcosm for multi-directional inter- and intra-kingdom horizontal gene transfer that has facilitated the evolution, selection, and adaptation of numerous microbes not only to evade predation but also to reside and proliferate within free-living amoebae (7, 17, 18). The long-term co-evolution within predatory amoebae has enhanced the pathogenic potential of many microbes to evade degradation and to reside and proliferate within mammalian cells, leading to life-threatening infections (19–21). This may not be surprising considering that various eukaryotic processes are highly conserved through evolution, including phagocytosis, vesicle traffic, endosomal-lysosomal degradation, ubiquitination, and various nuclear processes (15, 16). In addition, phagocytosis is considered a prerequisite for mitochondrion endosymbiosis and, therefore, a key component for eukaryogenesis (22).

**Fig 1:**
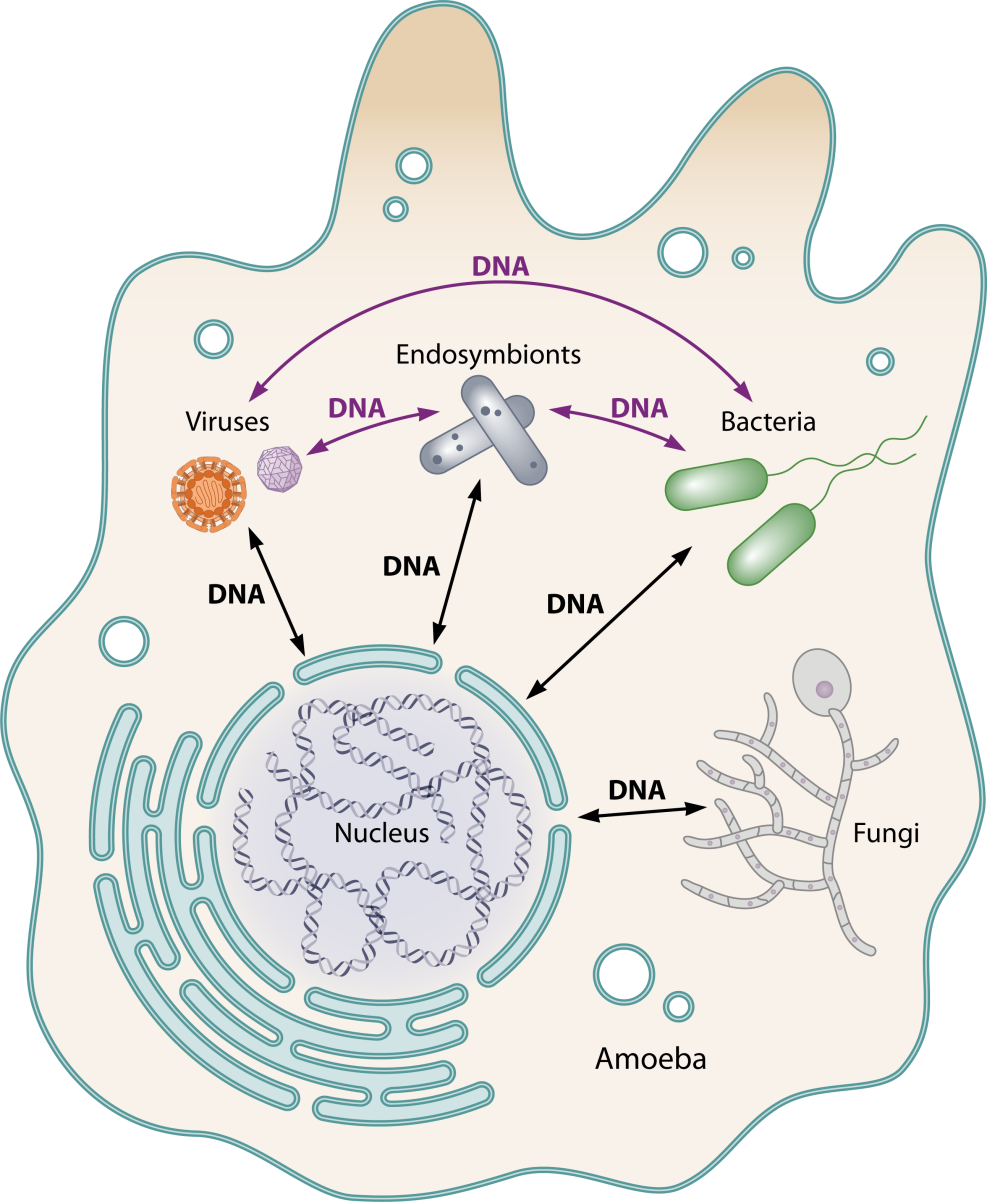
Multi-directional horizontal gene transfer drives the evolution of intracellular microbes with their amoebae hosts. As a result of the close association of microbes with protozoa, there has been tremendous multi-directional HGT that includes inter-kingdom between amoebae and the various intracellular microbial inhabitants as well as intra-kingdom HGT between various intra-amoeba microbial residents, endosymbiont, and amoebae mimivirues. This represents an immense and complex genetic melting pot that has driven the evolution and selection of numerous microbes.

Co-evolution with amoebae contributes to evolutionary adaptation and the ability of microbial pathogens to survive and replicate in human cells. However, it is also likely that rounds of selection through intermediate and multi-cellular hosts have occurred before infection ever happened to humans. In addition, many amoebae harbor a diverse range of obligate intracellular bacteria and endosymbionts that are highly adapted to the intracellular environment within amoebae (23–27). Single or simultaneous residence of intracellular microbes, endosymbionts, and giant viruses within free-living amoebae represent a genetic melting pot of multi-directional intra- and inter-kingdom horizontal gene transfer (Fig. 1) (7, 17, 18, 28).

Phylogenetic studies have shown that both *Coxiella* and *Rickettssia* share an evolutionary common ancestor with *Legionella*, and the three of them diverged from their common ancestor about 2 billion years ago (2). All of these three intracellular pathogenic bacteria harbor the Dot/Icm type IV secretion system, in addition to two of the nine core effectors that are present in all *Legionella* species (29–31). In addition, *Legionellae*, *Chlamydiae,* and *Rickettssia* genera have ancient co-evolution with their amoeba eukaryotic hosts (2, 32). Therefore, it is not surprising that several members of these bacterial genera are capable of invasion and replication within human cells to cause disease. In addition to these genera, many other bacterial pathogens including *Mycobacteria* spp, *Francisella tularenses*, *Escherichia coli*, *Campylobacter jejuni*, *Aliarcobacter butzleri*, *Pseudomonas aeruginosa*, *Salmonella Typhimurium,* and *Rhodococcus equi* in addition to fungi and *Acanthamoeba* giant viruses have transient and intimate interactions with amoebae. However, some microorganisms may have evolved to survive and replicate within protozoa without harming the host.

The immense diversity of unicellular protists represents most of the genetic, cellular, and biochemical diversity of eukaryotes (1). Our knowledge of pathogenic microbes-protists interaction is very limited. However, our current knowledge provides a remarkable insight into the evolution of pathogenic microbes through co-evolution and adaptation to unicellular eukaryotes as the training grounds for their subsequent capacity to infect mammals. The training grounds are where the inter- and intra-kingdom multi-directional horizontal transfer occurs and is a fascinating aspect of evolutionary biology. The immense number of eukaryotic-like genes in the pathogenic microbe and their original transfer from unicellular eukaryotes with their intracellular microbial residents is at the crux of pathogenic evolution and the emergence of numerous microbes infectious to mammals.

In 2005, we introduced, what was then considered a rather provocative, the concept that amoebae species represent a “training ground” for the evolution of pathogenic bacteria, as a new paradigm (5, 10, 33). Since then, there has been a quantum leap in our understanding of microbial adaptation to residence within predatory amoebae. However, our current knowledge of this complex co-evolutionary relationship remains the tip of the iceberg. In this review, we discuss our latest knowledge of the interactions of amoebae with endosymbionts, bacteria, fungi, and amoeba mimiviruses/giant viruses (Table 1). We discuss the role of amoebae as a unique microcosm of a genetic melting pot for multi-directional inter- and intra-kingdom horizontal gene transfer, which has been and continues to be, a remarkable “training ground” for the evolution and selection of specific microbial pathogens with capabilities to infect the more evolved mammalian host. These pathogenic capabilities in many microbes are mediated by microbial pathogenic factors that have been acquired, selected, and have evolved to target highly conserved eukaryotic processes, such as phagocytosis, vesicle traffic, ubiquitination, and nuclear processes (34).

**TABLE 1:** Summary of microbes associated with host amoebae species

Microorganism	Host amoebae
Symbionts	
*Criblamydia sequanensis*	*Acanthamoeba castellanii*
*Occultobacter vannellae*	*Vannella sp*. strain A1
*Berkiella aquae*	*Acanthamoeba*
*Nucleophilum amoebae*	*Amoebozoe sp*.
*Candidatus amoebophilus asiaticus*	*Acanthamoeba*
*Neochlamydia S13*	*Acanthamoeba castellanii*
*Burkholderia agricolaris*	*Dictyostelium discoideum*
*Burkholderia hayleyella*	*Dictyostelium discoideum*
*Legionella jeonii*	*Amoeba proteus*
*Neochlamydia hartmannellae*	*Vermamoeba vermiformis*
Obligate intracellular pathogens	
*Chlamydia sp*.	*Acanthamoeba sp*., *Vermaboeba sp*. and *Naegleria sp*.
*Rickettsia sp*.	*Acanthamoeba sp*.
*Coxiella sp*.	*Acanthamoeba sp*.
*Bartonella sp*.	*Acanthamoeba sp*.
*Legionella*	
*Legionella spp*	*Amoebozoa, Percolozoa, Ciliophora*
*Mycobacteria*	
*Mycobacterium avium* complex	*Acanthamoeba sp*., *Vermamoeba sp*., *Echinamoeba sp., Naegleria sp,*
*Mycobacterium marinum*	*Acanthamoeba sp*.
*Mycobacterium kansasii*	*Acanthamoeba sp*.
*Mycobacterium scrofulaceum*	*Acanthamoeba sp*.
*Mycobacterium chelonae*	*Acanthamoeba sp*.
*Mycobacterium fortuitum*	*Acanthamoeba sp*.
Other bacterial pathogens	
*Francisella tularensis*	*Acanthamoeba sp., Vermamoeba vermiformis*
*Francisella novicida*	*Acanthamoeba sp., Vermamoeba vermiformis, Dictyostelium discoideum*
*Francisella philomirigia*	*Acanthamoeba sp., Vermamoeba vermiformis*
*Rhodococcus equi*	*Acanthamoeba sp*.
*Campylobacter pylori*	*Acanthamoeba sp*.
*Aliarcobacter butzleri*	*Acanthamoeba sp*.
*S*. Typhimurium	*Dictyostelium discoideum*
*Vibrio cholerae*	*Acanthamoeba sp*.
*Pseudomonas aeruginosa*	*Acanthamoeba sp*.
Fungi	
*Torula famata*	*Acanthamoeba sp*.
*Candida sp*.	*Acanthamoeba sp., Vermamoeba vermiformis*
*Cryptococcus sp*.	*Acanthamoeba sp., Dictyostelium discoideum*
*Histoplasma capsulatum*	*Acanthamoeba sp*.
*Fusarium sp*.	*Acanthamoeba sp*.
*Aspergillus fumigatus*	*Acanthamoeba sp., Dictyostelium discoideum, Entamoeba histolytica*
Giant Viruses	
Mimiviruses	*Acanthamoeba sp*
Marseilleviruses	*Acanthamoeba sp*
Pandoraviruses	*Acanthamoeba sp*
Pithoviruses	*Acanthamoeba sp*
Faustoviruses	*Vermamoeba vermiformis*

## SYMBIONTS OF AMOEBAE AND THEIR ACQUISITION OF EUKARYOTIC GENES

Free-living amoebae and ciliates are ubiquitous in soil and aquatic environments, where they graze on bacteria, fungi, and algae. However, amoebae predation on microbes represents a strong selective force that has driven the evolution of microbial species that can resist or survive within amoebae hosts (17). In addition to the transient intracellular associations with amoebae, some of the microbial prey of amoeba have evolved into endosymbionts or ectosymbionts (23, 24). Most bacterial symbionts associated with amoebae are endosymbionts that are localized in the cytoplasm or nucleus of their host. Some are either free in the cytosol, such as *Criblamydia sequanensis* in *Acanthamoeba castellanii* (35, 36), or surrounded by host-derived membranes in case of *Occultobacter vannellae* in *Vannella sp*. strain A1 (36, 37). Some symbionts like *Berkiella aquae* and *Nucleophilum amoebae* are localized to the nucleus of *Acanthamoeba* or the perinuclear space of *Amoebozoe sp*., respectively (36). It is expected that nuclear residence of endosymbionts would enhance genetic exchange with the amoebae host. Remarkably, over 25% of *Acanthamoebae* species, which represent a highly abundant genera of amoebae, harbor endosymbionts (25–27). Moreover, at least one-third of wild *Dictyostelium discoideum* carry bacteria both intracellularly and extracellularly in their fruiting bodies (38). The most common symbionts in amoebae are affiliated with *Proteobacteria, Bacteroidetes*, *Chlamydiae*, *Firmicutes,* and *Cyanobacteria* evolutionary lineages (25–27). However, some less known bacterial phyla Candidatus *Dependentiae*, as well as unclassified and novel bacterial lineages, have been reported as symbionts of amoebae (24, 39). The amoebae symbiont *Candidatus amoebophilus asiaticus* encodes ~130 proteins (8% of the genome) with proteins containing eukaryotic domains and repeats such ANK repeats, TPR/Sel-1 repeats, LRR repeats, or F-box and U-box domains (27). In addition, the eukaryotic-like protein AnkB/Lpg2416 of *Legionella* spp (40–43) has been shown to originate from a mimivirus of *Acanthamoeba polyphaga* (44, 45). These findings clearly indicate that the encoding genes for these proteins have most likely been acquired from amoeba hosts and their intracellular residents rather than through convergent evolution (7, 34, 44, 46).

The evolution and stable establishment of endosymbionts within amoeba have diverse outcomes on their amoebae hosts, such as protection against infection by other microbes. For example, the *Neochlamydia S13* endosymbiont protects the *Acanthamoeba castellanii* host from infection by *Legionella pneumophila* (47). *Burkholderia (β-Proteobacteria*) can initiate a stable association with *Dictyostelium discoideum* in its two forms, vegetative and spore forms (48, 49). *B. agricolaris* and *B. hayleyella* benefit the amoebae in food-scarce conditions. When bacterial prey is depleted, *D. discoideum* disperses spores containing both *Burkholderia* and prey bacteria, seeding a garden of preferred food species for germinating spores to feed upon (38).

Some bacterial symbionts have no detectable positive or negative impact, at least so far, on their amoebae host, such as *Legionella jeonii* (γ-Proteobacteria) in *Amoeba proteus* (50); and *Amoebophilus asiaticus* (Bacteroidetes) in *Acanthamoeba spp* (27). However, some endosymbionts become pathogenic to their host (17). For example, infection of *Vermamoeba vermiformis* with *Neochlamydia hartmannellae* bacteria prevents cyst formation and subsequent amoebae lysis (51). It is likely that the establishment of endosymbiosis constitutes one of the initial major evolutionary steps and is the foundation for subsequent evolution and adaptation to microbial residence within predatory amoebae.

While many examples of bacteria-amoebae association have been studied, the vast diversity and complexity of amoebae-associated microbe interaction is poorly understood, and the number of symbionts is likely to be highly underestimated due to different factors. Most studies have focused on limited amoebae species within the *Amoebozoa* group including *Acanthamoeba* and *Dictyostelium,* which does not account for the vast diversity of the groups. However, it has been feasible to establish axenic and clean cultures from these two amoebae, but there are major difficulties in establishing axenic cultures of free-living amoebae from environmental samples. As a result, there is a relative paucity of genomic information and a lack of biochemical and cellular tools for most species of amoebae (17). Overcoming those limitations will reveal the impact of amoebae predation on symbionts, their subsequent evolution to become the predators of amoebae, and their subsequent evolution journey to infect mammalian cells.

## RESIDENCE AND CO-EVOLUTION OF *CHLAMYDIAE* AND *CHLAMYDIAE*-LIKE ORGANISMS WITHIN AMOEBAE

Obligate intracellular bacteria of free-living amoebae belong to the class of α- and β-proteobacteria that diverged into pathogenic and environmental chlamydiae 0.7–1.4 billion years ago. *Chlamydiae* are obligate intracellular pathogens of a wide range of eukaryotic cells including amoebae (52). Amoebae, particularly *Acanthamoeba spp*, serve as major environmental reservoirs of *Chlamydia spp* or *Chlamydia*-like bacteria in addition to having a role in their life cycle, ecology, virulence, and evolution (53). Remarkably, over 25% of *Acanthamoebae* species harbor endosymbionts (25–27).

Reconstruction of the genome of the last common ancestor of all known *Chlamydiae* showed that the *Chlamydial* ancestor infecting amoebae evolved later to endosymbiont-*Chlamydiae* after gaining many metabolic genes (32, 54). The *Chlamydial* ancestor has acquired and evolved all the genes required for an endosymbiotic lifestyle, as well some genes required for pathogenesis in higher eukaryotes, as some of these genes target mitochondria (55, 56). For example, the last common *Chlamydial* ancestor harbors type III secretion system (T3SS) encoding genes, which is conserved among *Chlamydia*-like bacteria (*P. acanthamoebae, S. negevensis*) and pathogenic *Chlamydiae,* and is essential for survival and proliferation of *Chlamydia* in the host cell (32, 53, 55). Adaptation and acquisition of effectors that modulate eukaryotic processes have likely contributed to continued evolution of the pathogen to infect humans (57–59). As discussed later, this is reminiscent of the T4SS of *Legionella*, and its role in early co-evolution and adaptation to unicellular eukaryotes and subsequent expansion to the human host (2).

Genome analyses provide clear clues about the evolution of Chlamydiae and its co-evolution with amoebae. *Chlamydiae* that infect mammals have smaller genomes compared to amoebae-associated *Chlamydiae*. The genome sizes of *P. amoebophila* and *W. chondrophila* are 2.4 Mbp and 2.1 Mbp, respectively, whereas the genome sizes of the human pathogens, *C. trachomatis* and *C. pneumoniae* are about half the size of 1.0 Mbp and 1.2 Mbp, respectively (60). This divergence in genomic size represents a trend that the bacterial genome is larger when there is a stronger dependency on the amoeba host (61, 62).

Free-living amoebae serve as a genetic melting pot for intra- and inter-kingdom horizontal gene transfer among intracellular microorganisms including *Chlamydiae* (10). It should not be surprising that amoebae-associated *Chlamydiae* have acquired numerous eukaryotic genes *via* horizontal transfer (HGT) from amoeba hosts, intra-amoeba prey microbes, endosymbionts, and amoeba Mimiviridae viruses (32, 53, 63, 64). In general, a large number of eukaryotic-like proteins or domains and eukaryotic tandem repeat-containing proteins are present in environmental chlamydiae and legionellaea (27). An example of eukaryotic proteins in environmental *Chlamydia* is the presence of 120 F-box proteins involved in ubiquitination (46, 65, 66). The leucine-rich repeats (LRR), Ankyrin repeat (ANK)-containing proteins, and the TPR repeats-containing proteins are very abundant in environmental *Chlamydia* vs pathogenic species (29, 30, 67). Other eukaryotic tandem repeat-containing proteins present in environmental Chlamydiae and Legionellaea include the repeats WD40, HEAT, RCC1, PPR, MORN, and Sel-1 repeats (65, 66, 68).

Lateral gene transfer between amoeba and Chlamydia and amoebae giant viruses has been documented (52), and plasmids may contribute to the gene transfer (69). Gene duplication in intracellular bacteria, such as *Chlamdia* and *Legionella*, is more frequent due to low genomic GC content of 42% and 39%, respectively (7, 34). Moreover, the *Chlamydia* ancestor evolved through gene loss. For example, the *Chlamydial* ancestor evolved through the loss of genes for amino acid biosynthesis (histidine, arginine, tryptophan, methionine, valine, leucine, isoleucine, phenylalanine, threonine, and purine) and maintained set of amino acid and oligopeptide transporters encoding genes to acquire amino acids from external sources (32). Moreover, conjugation and recombination have also been reported in amoebae-associated *Chlamydiae* (32, 63, 70, 71).

While the amoebae host range of different environmental Chlamydiae has not been extensively studied, infection of *Acanthamoeba* spp., *Vermaboeba* spp., and *Naegleria* spp. has been recorded (27, 53, 72). Several studies have described organisms closely related to *Chlamydiae* (*Chlamydia*-like bacteria) as symbionts of amoebae and various eukaryotic hosts (51, 70, 72–75). Like members of the *Chlamydia* genus, *Chlamydiae*-like bacteria have an obligate intracellular lifestyle (27, 53, 76), and share the unique *Chlamydial* developmental cycle consisting of an infectious extracellular elementary body (EB) and an intracellular replicative reticulate body (RB) (53, 77). The presence of intracellular *Chlamydia-*like bacteria within amoebae has divergent outcomes on the amoeba host, depending on the *Chlamydia-like spp.,* amoebae species, and environmental conditions. They can inhibit the entry of other bacteria into amoebae and compete more efficiently for nutrients with other intra-amoebae bacteria (47, 78). In terms of the effect on amoeba hosts, the growth of the amoeba host can be either enhanced or reduced (76, 79). Importantly, several species of *Chlamydia*-like bacteria such as *Waddlia chondrophila* and *Parachlamydia acanthamoebae* are potential emerging pathogens (80–83).

It is clear that many Chlamydia-like species have not completely evolved to expand the host range to mammals. As the process of evolution and multi-directional horizontal gene transfer within the genetic melting pot continues, it is likely that amoeba will continue to be a major training grounds for the evolution, selection, and adaptation of *Chlamydia*-like organisms to the intracellular life within phagocytic cells, which would facilitate expanding the host range to more evolved multicellular hosts.

## EVOLUTION AND DIVERGENCE OF *LEGIONELLALES, COXIELLA,* AND *PISCIRICKETTSIA* FROM A COMMON ANCESTOR

Bacteria adapting to living in a host cell caused the most salient events in the evolution of eukaryotic cells, namely the seminal fusion with an archaeon, and the emergence of both mitochondrion and chloroplast (84). A bacterial clade that may hold the key to understanding these events is the deep-branching γ-proteobacteria order *Legionellales*. The ancestors of the order *Legionellales* include the last common ancestor of *Legionellales, Coxiellaceae*, and the last *Legionellales/Piscirickettsia* common ancestor (LLPCA) (2). It is estimated that the last free-living ancestor of *Legionellales* existed at approximately 1.98 Ga LLPCA, whereas the first host-adapted LLPCA lineage existed approximately at 1.89 Ga. This implies that the host adaptation event that created *Legionellales* occurred almost ~2 Ga (2).

Phylogenetic studies have indicated that the Dot/Icm type IV translocation system and two of its translocated nine core effectors (AnkH and MavN) are present in all *Legionella* species as well as *Coxiella* and *Rickettssia* (2). These have been acquired by the bacteria during very early events of bacterial evolution and adaptation of the LLPCA to unicellular eukaryotic hosts (30, 85) that are estimated to have occurred almost ~2 Ga (2).

## THE IMMENSE AND DIVERSE TOOLBOX OF EFFECTORS IN THE *LEGIONELLA* GENUS

Upon inhalation of *L. pneumophila*-contaminated aerosols by humans, the organism proliferates within alveolar macrophages causing pneumonia designated as Legionnaires’ disease (86–88). However, *Legionella pneumophila* is an environmental bacterium that proliferates within a wide range of unicellular eukaryotes as its natural aquatic hosts spanning multiple phyla, from Amoebozoa (amoebae) to Percolozoa (excavates) to Ciliophora (ciliated protozoa) and 20–30 known species of amoebae (10, 89–91). With the development of anthropogenic water systems and management and the exposure to contaminated environmental aerosols, humans can serve as an accidental dead-end host of this intracellular bacterial pathogen (92), since there is no person-to-person transmission (93). This indicates that bacterial ecology and its association with amoebae are critical elements for bacterial ecology and its transmission to humans (94). This is supported by various findings that protozoa play major roles in the infection of human macrophages by *L. pneumophila* (10). Upon intracellular replication within protozoa, *L. pneumophila* exhibits a dramatic increase in resistance to harsh conditions including high acidity, temperature, high osmolarity, chemical disinfection, and biocides (10, 90). Importantly, upon their egress from amoebae, *L. pneumophila* exhibits enhanced infectivity for mammalian cells *in vitro* (10). Viable but non-culturable *L. pneumophila* can be resuscitated, even after chlorination, by co-culture with *Acanthamoeba* (95). Thus, the *L. pneumophila*-amoebae interaction is central to bacterial ecology, transmission, and infectivity in humans.

The ancient co-evolution and adaptation of legionellae to a broad range of protozoan hosts has been largely shaped by multi-directional horizontal gene transfer (HGT) including intra- and inter-kingdom, intra-species, and inter-species over ~2 billion years (2, 29, 96–100). This melting pot of long-term HGT to *Legionella* spp. within amoebae has likely come from the amoebae host and its endosymbionts, other intra-amoeba bacteria, fungi, and mimiviruses/giant viruses of amoebae (29, 30, 73, 96, 101). For example, the eukaryotic-like protein AnkB/Lpg2416 effector *Legionella* spp (40–43) has originated from a mimivirus of *Acanthamoeba polyphaga* (44, 45). It is evident that inter-kingdom HGT within the amoebae host has been the major driving force for the evolution of *Legionella* and its adaptation to the intracellular life within protozoa and expansion of its subsequent host range to humans.

The exploitation of conserved eukaryotic host processes by *Legionella* is very evident from its indistinguishable intracellular trafficking within the evolutionarily distant hosts. Following phagocytosis by amoebae or human macrophages, the phagosome harboring *L. pneumophila* evades the evolutionarily conserved endosomal-lysosomal degradation pathway and intercepts early secretory vesicles to become an ER-derived vacuole, designated as the *Legionella*-containing vacuole (LCV), and this process is indistinguishable in evolutionarily distant host cells (102–107). The unique biogenesis of the LCV and modulation of numerous conserved eukaryotic processes within amoebae and human macrophages is mediated by the Dot/Icm type IV secretion system that injects a plethora of 350–400 protein effectors into the host cells (30, 108), and many are targeted to the host nucleus (109). The numerous highly conserved eukaryotic processes such as phagocytosis, vesicle traffic, and various nuclear process, which are all targeted by the immense toolbox of effectors, have undoubtedly facilitated the infection of macrophages by *L. pneumophila*.

Among all known human pathogens, *L. pneumophila* has the largest repertoire of effectors, with around ~11% of the *L. pneumophila* genome encoding capacity dedicated to encoding effectors. The large number of *Legionella* effectors along with their redundancy illustrates a remarkable evolution and selection of this arsenal in the adaptation of *Legionella* to a wide variety of environmental hosts, as a “generalist” pathogen. Each of the ~60 species of *Legionella* has a specific set of effectors or “tools,” since only 32% of the genes found in the *Legionella* pangenome are strain specific, indicating the interaction and co-evolution of each *Legionella* species with distinct protozoan hosts in various environments around the globe (7, 29, 30, 34, 110). There is a large degree of plasticity and number of effectors among *L. pneumophila* clinical isolates, leading to distinct phenotypes of corresponding mutants in different strains. The variability of the effector toolbox is even more dramatic across *Legionella* species, with only 52 effectors in *L. adelaidensis* compared to the ~350 in *L. pneumophila* (29, 30). Considering the ancient 1–2 billion years of co-evolution of *Legionella* with diverse protozoan species, the toolbox of effectors is impacted by the cumulative acquisition and selection of the large repertoire of redundant effectors within a broad range of environmental hosts along with their intracellular transient and permanent residents microbes (10, 30, 91, 96, 98, 101, 111–114). It is likely that additional co-evolution and adaption to other multicellular eukaryotic hosts have been factors in the expansion of the host range and infection of the more evolved human macrophages.

The *Legionella* genus has at least 60 species that have been isolated from diverse aquatic and soil environments across the globe including hot springs and Antarctica (115–121). However, to date, other than *L. pneumophila,* only *L. longbeachae* has been studied in any detail. Remarkably, over 60% of the known *L. pneumophila* effectors are absent in the *L. longbeachae* genome, while it harbors over 50 unique effectors that are absent in *L. pneumophila* (110, 122).

*L. longbeachae*, like other members of the *Legionella* genus invades and replicates in environmental amoebae, but in comparison to *L. pneumophila*, *L. longbeachae* is found more commonly in potting soil rather than aquatic environments (123, 124). *L. longbeachae* evades lysosomal fusion in both the natural amoebae host and human macrophages and generates a non-acidified late endosome and ER-derived LCV, which is different from the LCV harboring *L. pneumophila* that largely evades the endosomal-lysosomal pathway (123, 125, 126).

## EUKARYOTIC ORIGIN OF AMOEBA-ADAPTED EFFECTORS OF *L. PNEUMOPHILA* AND THEIR DISPENSABILITY WITHIN MACROPHAGES

The inter-kingdom HGT from protozoa to *Legionella* spp. is very evident from genomic and metagenomics analyses (7, 21, 29, 30, 127). The genes encoding many of these effectors with eukaryotic motifs/domains or eukaryotic-like proteins possess higher GC content than the rest of the *Legionella* genomes (29). By contrast, the GC content of the nine core effectors present in all *Legionella* spp. is similar to the rest of the genome indicating a more ancient acquisition of these genes during the early stages of interaction with protozoan hosts (31). The high degree of HGT and genomic plasticity of *L. pneumophila* is consistent with its competency for DNA uptake by natural transformation through the type IV pili as well as conjugation through T4ASS and T4BSS systems (128).

Among the 18,000 effectors in the *Legionella* genus, the *Legionella* eukaryotic-like effectors contain ~140 different eukaryotic-like domains or domain combinations many of which are uniquely present in protozoan proteins but not humans, indicating their origin from the amoebae host (7, 21, 29, 30, 127). Importantly, 184 *Legionella* genes are predicted to encode eukaryotic-like small GTPases (7, 29, 34), and 71 of them are predicted to encode eukaryotic-like Rab GTPases with high similarity to Rab GTPases of protozoa, such as *Entamoeba* or *Tetrahymena*e.

It may not be surprising that most of the ~350 Dot/Icm-injected effectors of *L. pneumophila* are dispensable for infection of macrophages (106). Remarkably, even the simultaneous elimination of ~60 effectors has no significant effect on the growth of *L. pneumophila* within mouse macrophages (106, 114, 129) but there are few exceptions where the deletion of a family of redundant effectors have a modest impact on the growth of *L. pneumophila* within macrophages (130–132). However, even when redundant family effectors are deleted, there is no detectable role for the majority of the families of redundant effectors in the infection of macrophages (5, 29, 30, 98, 106, 113, 114, 129). Redundancy of some of the effectors is thought to be one factor for the lack of a detectable role of the effectors in bacterial proliferation within macrophages (112).

## FATE OF AMOEBAE-ADAPTED EFFECTORS OF *LEGIONELLA* WITHIN MACROPHAGES

**Fig 2:**
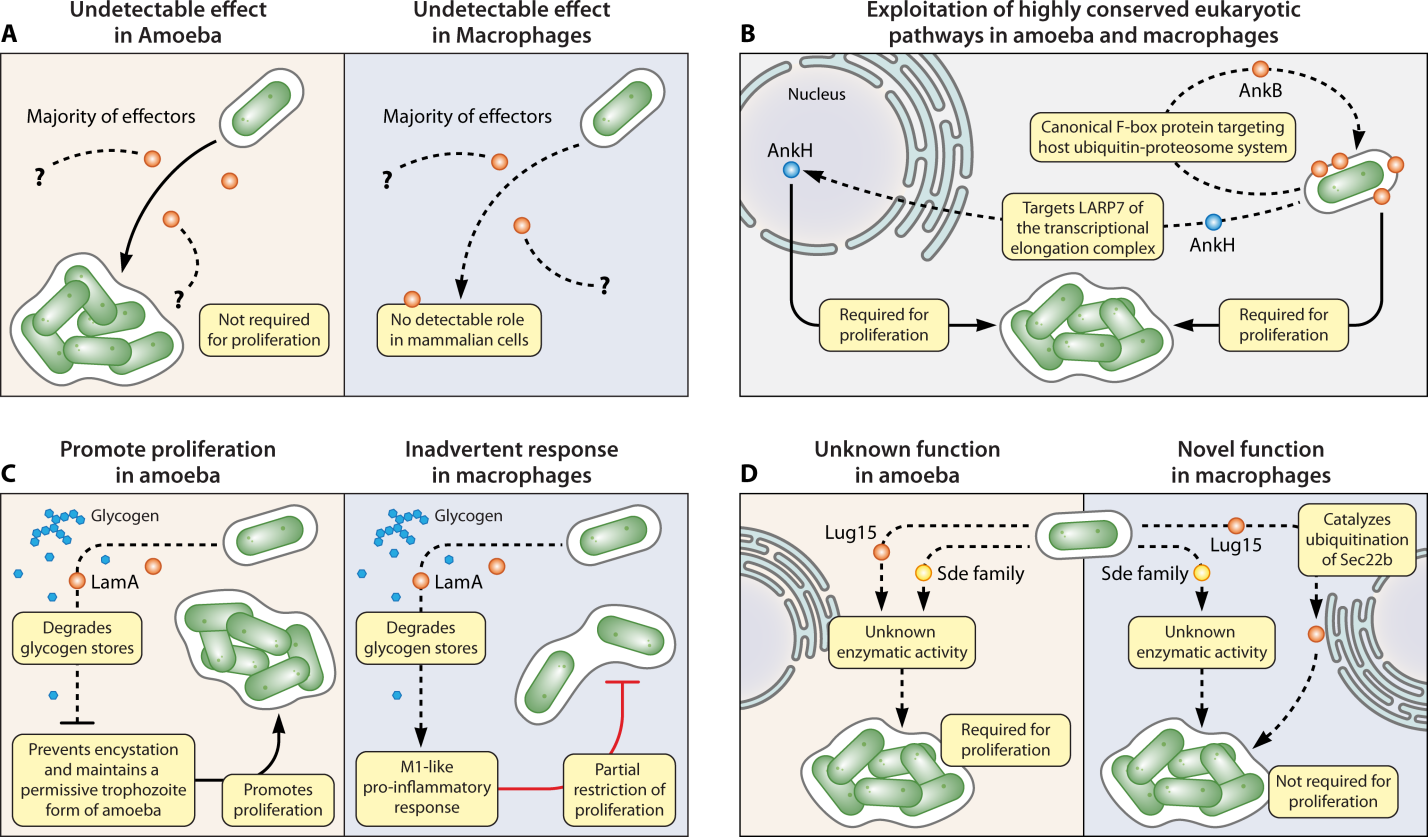
The fate of amoebae-adapted effectors of *Legionella* in the natural amoebae host and macrophages and their potential novel biochemical functions. The molecular toolbox of *L. pneumophila* effectors has evolved to modulate diverse processes present within amoebae hosts and subsequently can have various distinct outcomes when injected into human macrophages. (**A**) Most effectors are not required for pathogen proliferation within amoebae or mammalian cells. This can be simply due to the facts that most effectors have not been tested in various protozoan species and are likely to have an effect in certain protozoan hosts. (**B**) An injected effector interacts with a highly conserved eukaryotic target present in amoebae and mammalian cells and is required for intracellular bacterial replication. For example, the AnkB and AnkH effectors that target the host ubiquitin-proteasome system and the LARP7 component of the transcriptional elongation complex, respectively, in both amoebae and human macrophages. (**C**) An amoebae-adapted effector interacts with the same target in the two evolutionarily distant hosts, or by accident to a distinct mammalian target, leading to an accidental response in the mammalian host. For example, the LamA amylase effector depletes glycogen stores in *Acanthamoebae*, interferes with the encystation of amoebae to enable replication of *L. pneumophila* in the permissive trophozoite form of the amoeba, while the cyst form is non-permissive. LamA also depletes glycogen in human macrophages, but this inadvertently triggers an M1 pro-inflammatory response that partially restricts *L. pneumophila* replication. (**D**) Amoebae-adapted effectors may harbor novel enzymatic functions previously unknown or absent in mammalian cells. For example, the Sde family of effectors catalyze novel phosphoribosyl-ubiquitination of host proteins by a novel single E1/E2 ligase-independent enzyme, which has not been described in mammalian cells, but it is not known whether a similar ubiquitination is exhibited in protozoa. The Lug15 effector is a structurally novel E3 ubiquitin ligase that triggers canonical ubiquitination of Sec22b of mammals but not known whether a similar ubiquitination is exhibited in protozoan hosts. These novel structural or catalytic activities of various effectors not known in mammals may be amoebae-specific biochemical functions, acquired from the amoebae hosts, or may be exhibited but not known yet in mammals.

**Fig 3:**
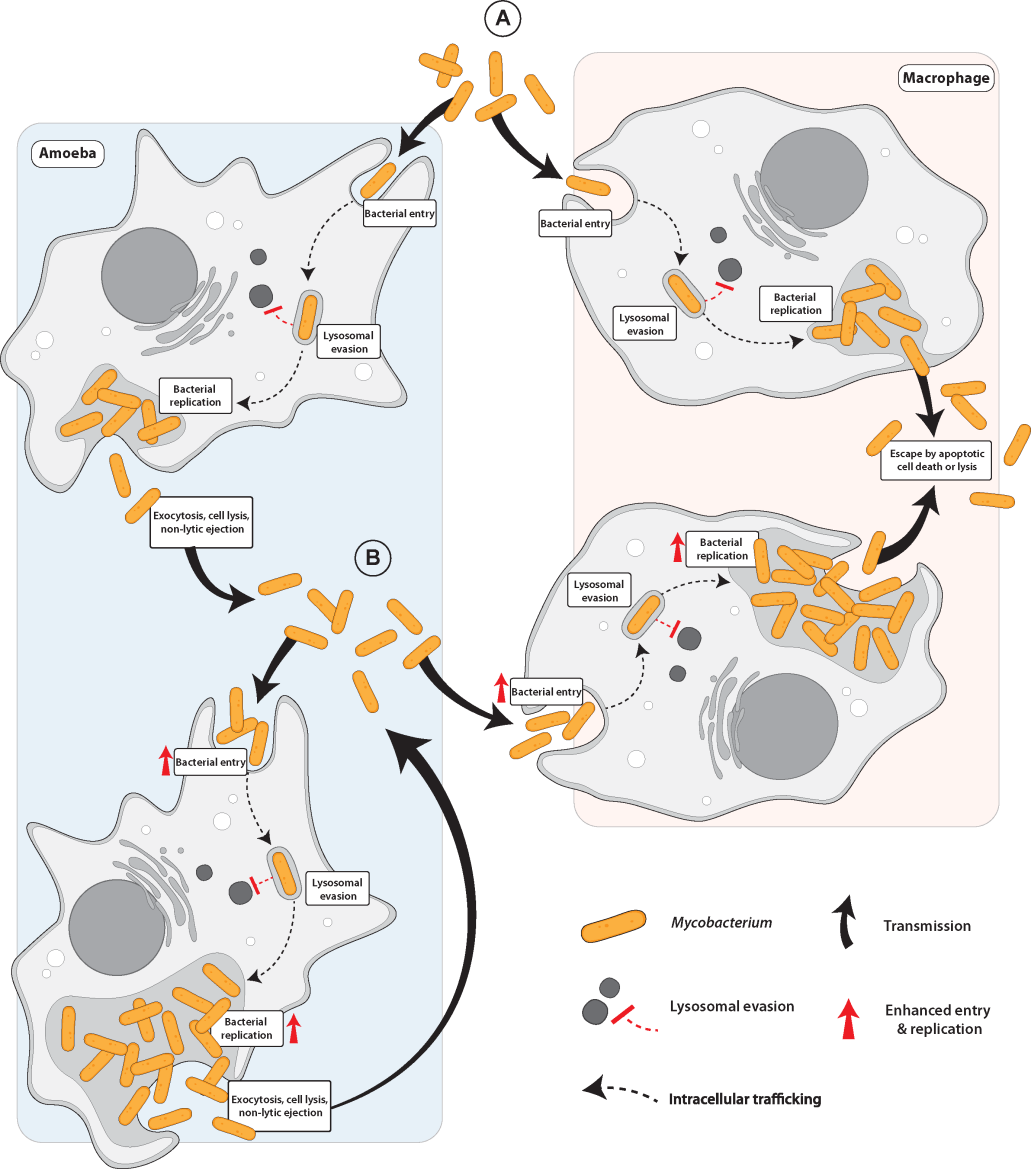
Amoebae promote expansion and adaptation of *Mycobacterium* to the mammalian host. The epidemiology and ecology of pathogenic *Mycobacterium* exhibit striking similarities to *L. pneumophila* and are the result of amoebae pre-adapting *Mycobacterium* to the mammalian host. (**A**) After phagocytosis by amoebae or macrophages, the *Mycobacterium* phagosome evades lysosomal fusion, and the bacteria replicate prior to release back into the surrounding environment by exocytosis, ejection, or host cell lysis. (**B**) Following release from the original amoeba host, amoeba-grown *Mycobacterium* demonstrates enhanced virulence and entry into amoebae and macrophages compared to *Mycobacterium* cultured without amoeba. In addition, the enhanced proliferation of amoeba-grown *Mycobacterium* promotes host-cell lysis and pathogen dissemination throughout the environment and mammalian host.

It is more likely that a large number of the effectors are protozoan hosts-adapted effectors that may not have a target in macrophages and can result in various outcomes in mammalian cells compared to a protozoa-specific effect. Once protozoan host-adapted effectors of *Legionella*, or other amoeba-adapted pathogenic bacteria, are injected into macrophages, they may result in at least three distinct outcomes (Fig. 2 and 3): First, the majority of *L. pneumophila* effectors lack any detectable role in mammalian cells. This may be due to the lack of mammalian targets for amoebae-adapted effectors in human cells or to compensation by redundant or structurally distinct effectors.

The second possible outcome upon injection of protozoa-adapted effectors into macrophages is that the effector exploits a highly conserved eukaryotic process. In this case, the effector is likely to be required for the infection of evolutionarily distant hosts (114), such as the AnkB and AnkH effectors. The AnkB effector functions as a eukaryotic canonical F-box protein that is farnesylated by the host farnesylation machinery that anchors it to the membrane of the pathogen-containing vacuole where it exploits the highly conserved eukaryotic ubiquitin-proteasome system (42, 43) and is indispensable for bacterial proliferation of within the evolutionarily distant hosts (41–43). The injected AnkH effector of *L. pneumophila* is sorted to the host nucleus where its interacts with the highly conserved LARP7 subunit component of the 7SK snRNP transcriptional elongation complex and is required for proliferation in the two evolutionarily distant hosts (133).

The third possible outcome upon injection of protozoa-adapted effectors into macrophages is that macrophages may respond through either promotion or restriction of bacterial replication, which are considered inadvertent macrophage responses to protozoan host-adapted effectors (114, 134). The amoebae host-adapted *Legionella* amylase (LamA) effector interferes with the encystation of *Acanthamoeba* to maintain it in the trophozoite form, which is the permissive form. This is mediated by the rapid LamA-mediated degradation of *Acanthamoeba* glycogen stores, which is the main resource for amoeba to synthesize the cellulose double-layer membrane of the cyst (134). However, when the amoeba-adapted LamA is injected into macrophages, rapid degradation of glycogen results in a rapid cytosolic hyper-glucose leading to an inadvertent M1-like pro-inflammatory response (134). This accidental pro-inflammatory response by macrophages triggers nutritional immunity through tryptophan degradation, leading to a partial restriction of intracellular proliferation of *L. pneumophila* within human macrophages (134). This paradoxical effect of LamA in the human host is more detrimental in neutrophils that respond to the LamA-dependent glycogen degradation and cytosolic hyper-glucose through spatial generation of reactive oxygen species within the LCV as well as fusion of the LCV to neutrophil granules, leading to a rapid degradation (within 15 minutes) of *L. pneumophila* (135). Therefore, LamA is a clear example of an amoebae host-adapted effector that has an unexpectedly paradoxical effect on the accidental human host.

It remains elusive why some effectors have novel eukaryotic novel biochemical functions not known to be present in macrophages, but it remains unknown whether these novel structural and biochemical functions are present in unicellular eukaryotes. The Lug15 effector and the SidE effector family of effectors have been shown to exhibit novel structural or enzymatic functions, respectively, not known to be present in mammalian cells (29, 30, 98, 113, 114). The Sde family of four redundant effectors has been shown during macrophage infection to protect the integrity of the LCV during the early stages of its establishment (136) through catalyzing novel phosphoribosyl-linked chemistry of ubiquitination catalyzed by a single enzyme that does not require ATP (137), which is not known to be found in mammalian cells. It is not known whether the Sde family exhibits a similar effect in protozoan hosts. The Sde family is likely to be amoeba-adapted effectors, since they are required for proliferation within *Acanthamoeba* and *D. discoideum* but not in mammalian macrophages (102, 137–145). Therefore, this novel ubiquitination activity is likely to be a protozoa-specific enzymatic activity that has been co-opted by *L. pneumophila* from its protozoan hosts (137). The Lug15 effector *L. pneumophila* is a novel E3 ubiquitin ligase with no structural homology to other E3 ligases but catalyzes canonical ubiquitination of mammalian Sec22b (136). It remains unknown whether Lug15 has similar structural similarities to protozoan E3 ubiquitin ligases, and whether it has any biochemical modulation of ubiquitination in various protozoan hosts. Similar observations have also been made for the type II secretion system of *L. pneumophila* and its secreted proteins, which are required for growth in amoebae hosts but not human macrophages (146–149).

Therefore, it is important for future studies that various protozoan hosts should be included side-by-side with macrophages to study the role of *L. pneumophila* effectors. Studies utilizing more protozoan species will also likely reveal novel functions of *L. pneumophila* effectors that modulate amoebae-specific processes absent in mammals such as novel ubiquitin ligases and GTPases.

*L. pneumophila* has been and will continue to be a remarkable biological tool to dissect various biological and biochemical eukaryotic processes and uncover various unknown biochemical activities that modify eukaryotic proteins. Interestingly, the RomA effector catalyzes H3K14 methylation of histones in macrophages and amoeba, which was not known to be present in mammalian cells (150, 151). However, due to this discovery, studies have later shown that mammals also exhibit H3K14 methylation of histones (152, 153). The novel ubiquitination of macrophage proteins by the Sde family of effectors may also be exhibited, but not know yet, by mammalian cells. As mentioned above, the paucity of research tools to study cell biology of amoeba along with limited genomic analyses of amoeba hosts will continue to be a challenge to advance our knowledge.

## THE AMOEBAE-ADAPTED OBLIGATE INTRACELLULAR *LEGIONELLA-*LIKE AMOEBAL PATHOGENS

Various bacteria that evade degradation by predatory amoeba are emerging human pathogens including the *Legionella***-**like amoebal pathogens (LLAPs), *Parachlamydia acanthamoebae*, *Simkania negevensis*, waterborne *Mycobacteria,* and *Bradyrhizobiaceae* (154). The LLAPs are primarily obligate intracellular bacteria that reside within amoebae (155–157). Their LLAP designation originates from their ability to infect and proliferate within amoebae, similar to other species of *Legionella,* and their phylogenetic closeness (155–158). Many of the LLAPs have been isolated from clinical specimens of pneumonia patients by co-culture with amoeba with little or no growth on artificial media (155–157). There is also serological evidence indicating causation of the LLAPs of cases of pneumonia (159). Many LLAP strains have been also isolated from environmental sites of transmission of Legionnaires’ disease and all the LLAPs are capable of causing pneumonia (154). Based on genomic and metagenomics analyses, most of the LLAPs have been classified into new *Legionella* species (154).

LLAP-1 has been classified into *Legionella drozanskii* sp. nov. The LLAP-3, -7, and -9 strains are members of the species *Legionella lytica* (158). LLAP-6 has been classified into *Legionella rowbothamii* sp. Nov (158). LLAP-10 has been classified into *L. Fallonii*. LLAP-12 has been classified into *Legionella drancourtii* sp. nov., and is highly related to LLAP4 and 11 (160). The obligate nature of the LLAPs suggests that they are at a different stage of evolution compared to other facultative intracellular species of *Legionella*, but may likely continue to evolve with protozoan hosts. Classification of some LLAPs remains to be determined but studies on this group of pathogens should shed further light on the evolution of *Legionella*-protozoa interaction and its infectivity to humans.

## INTERACTIONS OF OBLIGATE INTRACELLULAR BACTERIAL PATHOGENS WITH AMOEBAE

*Rickettsia, Coxiella*, and *Bartonella* are three obligate intracellular bacterial pathogens that interact with amoeba. *Rickettsiales* are capable of infecting a diverse range of eukaryotic hosts from protists to arthropods and mammals (161–163). These bacteria along with *Coxiella* and *Legionella* share a common ancestor that they diverged from and all harbor the Dot/Icm translocation system, as well as two of the nine core effectors of *Legionella* (2, 29, 30).

Phylogenic analyses of *Rickettsiales* resulted in the separation of bacterial members into the typhus group, spotted fever group, transitional group, *R. canadensis* group, and the *R. bellii* group (164–166). Phylogenic and genomic analyses suggest that *R. bellii* diverged from the other *Rickettsiales* prior to the typhus and spotted fever groups and possesses many genes with closer relation to other amoebae symbionts than other *Rickettsiales* (164–166). A likely explanation for this early divergence may be related to the ability of *R. bellii* to survive within environmental amoebae and undergoing multi-directional HGT within the amoebae host and its intracellular residents and preys, such as *L. pneumophila, Candidatus Protochlamydia amoebophila* endosymbionts, and mimiviruses (164). Amoebae endosymbionts of the *Rickettsiales* include *Rickettsiaceae*, *Anaplasmataceae*, *Holosporaceae*, and *Candidatus* Midichloriaceae (167, 168).

Genomic analysis of these endosymbionts has indicated recurring HGT between them and diverse amoeba-associated bacteria (33, 168, 169). In addition, many plasmids and genes included in HGT play an important role in amoeba-symbiont interactions involved in stress response, bacterial transport systems, antibiotic resistance, and bacterial virulence (168). Genomes of *Rickettsiales* strongly reflect host adaptation for modulating host cellular processes and establishing a niche for energy parasitism (167, 170). The α-proteobacterium *Rickettsia prowazekii*, which is considered the progenitor of the eukaryotic mitochondria, possesses higher numbers of eukaryotic-like proteins, indicating a high level of long-term inter-kingdom HGT (171, 172). Similar to *Legionella* and environmental *Chlamydia*, other bacteria that are able to survive and replicate within amoebae harbor large numbers of eukaryotic-like proteins or domains (27, 44, 171, 173, 174). Thus, *Rickettsiales* share similarities to *Legionella* and provide a solid example of ancestral inter- and intra-kingdom HGT within amoebae long before interactions of prokaryotes with multicellular eukaryotes.

*Coxiella burnetii*, originally categorized as a member of *Rickettsiales*, is an obligate intracellular pathogen that primarily infects arthropods along with mammalian macrophages and monocytes, but has been mainly studied within mammalian cell models (175, 176). However, few studies have revealed interactions between *C. burnetii* and amoebae, which is not surprising considering the ancestral relationship with *Legionella* (2) in having genes of eukaryotic origins (177).

Co-culture of *C. burnetti* with *A. castellani* results in extended survival and replication of *C. burnetii* within amoebae, along with an increased differentiation of *C. burnetii* into a highly pathogenic spore-like form (175). Like with *Legionella,* amoebae provides a replicative niche and protection of *C. burnetii* against environmental stressors, promoting transmission of *C. burnetii* in contaminated water sources (178–181). While further studies are needed to clarify in-depth the evolutionary relationship between *C. burnetii* and amoeba, the current information sheds some light on how amoebae may protect *C. burnetii* from environmental stressors, increasing bacterial pathogenicity and promoting transmission to mammals (182).

*Bartonella* species are obligate intracellular bacteria that have diverged from *Rickettsiales* and infect arthropods, erythrocytes, and mammalian endothelial cells (169, 176). Members of the *Bartonella* genus live an allopatric lifestyle with their host cells and rarely share an overlapping niche for genetic exchange with other bacteria (169, 183). However, phylogenetic analysis demonstrated the T4SS of *Bartonella* has been acquired through HGT from conjugative plasmids (184, 185). Due to similarities with the *Rickettsia* sex-pili appendages, phylogenetic studies suggest *Bartonella* species gained T4SS expression and conjugation capabilities through interactions with ancient bacterial species already possessing a conjugation system and plasmids (169). During co-culture with amoeba, *B. rattaustraliani* exhibits DNA exchange by conjugation with *R. radiobacter* and transfer of plasmids (169). Therefore, amoebae have likely played a role in pathogenic evolution of *Bartonella* through acquisition and transfer of conjugative plasmids to other bacteria co-inhabiting with the same amoebae host (169).

## THE DIVERGENT INTRACELLULAR LIFESTYLE OF *FRANCISELLA TULARENSIS* IN AMOEBAE AND MACROPHAGES

*Francisella tularensis* is a Gram-negative facultative intracellular bacterium and the causative agent of the zoonosis, tularemia (186). Since *Francisella* spp. are facultative intracellular pathogens ubiquitous in the environment, it is not surprising that *F. tularensis* subsp. *tularensis* invades and replicates within amoeba species (187–193). In nature, *F. tularensis* occurs in two cycles, terrestrial and aquatic and can survive environmental conditions for a long period of time (194). The bacterium has been isolated from more than 100 mammals, birds, cold-blooded animals, and arthropods (195). In addition, subsp. *novicida* and *philomiragia* also replicate in *A. castellanii* and *Vermamoeba vermiformis* (187, 188, 192, 196) (Table 1). The subspecies *novicida* and *noatunensis* also infect and replicate in *Dictyostelium discoideum* (189, 197).

Within the genus *Francisella*, only the three subspecies tularensis (type A), *holarctica* (type B), and *mediasiatica* (198) are of clinical importance causing infection in humans, with type A and B causing the more severe illnesses. Transmission of *F. tularensis* to humans can occur through bites from vectors (ticks, flies, or mosquitoes), improper handling of infected animals, contact with water bodies, and inhalation of contaminated aerosols (199, 200). Upon uptake by mammalian cells, *F. tularensis* delays acidification of the *Francisella*-containing vacuole (FCV) and evades phagosome-lysosome fusion (201–203), followed by rapid pathogen egress into the host cytosol, which is dependent on the VI secretion system (T6SS) (188, 204–206). In contrast to bacterial proliferation in the cytosol of mammalian macrophages, *F. tularensis* proliferates within membrane-bound vacuoles that evade lysosomal fusion in *A. castellanii* or *V. vermiformis* (188, 190, 207). Interestingly, in contrast to the growth of *L. pnumophila* within amoebae and its major effect in enhancing pathogenic properties (10), the growth of *F. novicida* in *A. castellanii* had no effect on virulence in mice compared with *in vitro*-grown *F. novicida* (196), but it is not known whether that affects infection of humans.

Although the *Francisella* pathogenicity island encoded-T6SS (204–206) plays an important role in intracellular trafficking and replication of *Francisella* species in mammalian cells, proliferation of the pathogen within amoebae is independent of the T6SS (208). The divergence in the requirement of the T6SS within mammalian and protozoan cells is perplexing and unexpected. It clearly indicates that the effectors translocated by the T6SS do not play a role in the adaptation of *Francisella* to amoebae hosts. The bacterial factors involved in adaptation to amoeba hosts remain unknown.

Several amoebae species, including *A. castellanii*, *A. polyphaga,* and *V. vermiformis* enhance survival of *F. novicida*, and *F. tularensis* (type A and type B) over a 10-day period, but neither their proliferation nor lysis of amoebae host cells is detected (193). Within *A. castellanii* cysts, *F. tularensis* type A survives for at least 3 weeks (187). Survival within amoebic cysts plays an important role in *F. tularensis* survival in the environment for longer periods (187), which could partially explain the transmission of tularemia through water.

After the proliferation of *F. tularensis* in amoebae, the bacteria exhibit longer survival in the environment, greater virulence, and resistance to antibiotics and disinfectants (209). However, compared to *in vitro* grown bacteria, *F. novicida* grown in amoebae are more sensitive to disinfection (210). These results suggest that amoebae can enhance the environmental survival of *Francisella* species, which is likely through physical protection and nutrient availability. Interestingly, amoebae supernatant reduces the biofilm formation of *F. philomiragia* (192) and enhances the proliferation of *F. tularensis* (211). These studies indicate that growth of some *Francisella* species is enhanced by the amoebae-conditioned medium. However, infection of amoebae with some clinical isolates of *F. tularensis* subsp. *tularensis* results in a decrease in the viability of intracellular bacteria (187), suggesting that intracellular growth in amoebae depends on the *Francisella* species but also the medium and protocols used in the study. The ongoing co-evolution of *Francisella* with amoeba species is likely to continue and will gradually select bacterial variants that are better adapted to the intracellular life within amoeba. It remains unclear whether the T6SS translocates eukaryotic-like substrates acquired through HGT.

## CO-EVOLUTION OF *MYCOBACTERIA* WITH AMOEBAE

Majority of mycobacterial species are environmental organisms commonly found in water, soil, and air (212–215). Among these are opportunistic pathogenic species associated with bacterial colonization of domestic and environmental water sources are the *Mycobacterium avium* complex, *M. marinum*, *M. kansasii*, *M. intracellulare, M. scrofulaceum*, *M. chelonae* complex, and *M. fortuitum* (213, 216–218). The similarities in the epidemiology and ecology of aquatic *Mycobacterium* species and *L. pneumophila* and their pulmonary infections of mammals (219, 220) have led some studies to determine whether interactions of *Mycobacterium* species with water-borne amoebae promotes the virulence and disease manifestation within the mammalian host (216, 221). However, *M. tuberculosis* seems to be highly adapted to humans, as evident from the manipulation of highly specific mammalian processes (221, 222).

*M. avium* complex, *M. leprae*, *M. marimun, M. kansasii, M. scrofulaceum*, *M. xenopi,* and *M. fortuitum* survive within amoebae, where the bacterium-containing phagosomes evade lysosomal fusion (216, 223, 224). In addition, these species withstand amoebae encystation while non-pathogenic species of *Mycobacterium* are degraded within encysted amoebae (216, 223, 224). Co-culture of *M. avium* with *A. castellanii* enhances bacterial entry into other amoebae cells, epithelial cells, and macrophages (225) (Fig. 3) (Table 1). Amoebae-grown *M. avium* exhibits enhanced proliferation and virulence in macrophage and mouse infection models (216, 225, 226). This is reminiscent of the enhanced pathogenesis of *L. pneumophila* after its invasion and growth within amoebae (227). Similar to *L. pneumophila*, lysosomal evasion within amoeba has likely facilitated the ability of various *Mycobacteria* species to evade lysosomal degradation within macrophages, as these eukaryotic processes are highly conserved through evolution (228). As an evidence for this, the *Mycobacterium*-containing phagosome within mammalian macrophages matures to an early endosome-like phagosome that is connected to early endosomal traffic (229–232).

Genomic analyses of *M. avium* identified a pathogenicity island (PI) encoding glycolipid biosynthesis genes and membrane proteins that are absent from *M. tuberculosis* and *M. paratuberculosis* (223, 233). This PI has been likely acquired by HGT from other environmental microorganisms within amoebae or extracellularly within biofilms (223, 234). The *M. avium* pathogenicity island (PI) is essential for infection of amoebae and macrophages (223, 235–237). Deletion of this PI of *M. avium* significantly reduces bacterial entry into both amoebae and macrophages, suggesting this PI may have been initially acquired to facilitate *M. avium* entry into amoebae which then carried over to macrophages (Fig. 3) (223). Therefore, water-borne *Mycobacterium* species have co-evolved with amoebae, similar to *L. pneumophila*, where they have adapted to evade lysosomal degradation and to proliferate within predatory environmental amoebae. It is likely that through HGT, *Mycobacteria* have been equipped with the tools to exploit conserved eukaryotic processes, such as the endosomal-lysosomal degradation pathway, that have facilitated infection of mammalian cells.

## CO-EVOLUTION AND ADAPTION OF *RHODOCOCCUS EQUI* WITH AMOEBAE

Although *Rhodococcus equi* is often found in dry soil, where it evades amoebae predation that could contribute to the overall survival and dissemination of *R. equi* in the environment (238). This bacterium is a facultative intracellular pathogen that replicates within mammalian macrophages causing pneumonia in young horses and is also an opportunistic pathogen of immunocompromised humans (239, 240). The survival and intracellular proliferation of virulent *R. equi* within phagocytes depend on the presence of an 80 kb virulence plasmid which encodes a family of virulence-associated proteins (Vap) (241, 242). The VapA protein plays a key role in the exclusion of the host vacuolar ATPase from the *R. equi*-containing vacuole and in permeabilization of lysosomes, resulting in a neutral lysosomal pH (243). The VapA protein plays a role in the evasion of degradation within macrophages and *A. castellanii* (238). It is possible that *trans*-effect of pH neutralization of various cellular vesicles and compartments by VapA has enabled the adaptation of other intra-amoebae bacteria to the intracellular life within amoeba co-inhabited with *R. equi*. Since VapA exerts a function related to the modulation of the function of eukaryotic vesicles, it is likely that VapA has been acquired through HGT during bacterial co-evolution within amoebae, and is clearly a major factor for evasion of lysosomal degradation in mammalian macrophages. It would be interesting to identify the host targets of VipA within macrophages and amoebae to determine the pathogenic evolutionary history of *R. equi*. This will provide insight into the co-evolution of *R. equi* with amoebae and the conserved eukaryotic processes exploited by the pathogen and its role in the host expansion to humans.

## ROLE OF AMOEBAE IN PATHOGENIC EVOLUTION OF GASTROINTESTINAL PATHOGENS

Many gastrointestinal bacterial pathogens that can survive in aquatic environments have been shown to interact with amoeba. In many cases, this bacteria-amoebae interaction has been shown to be important for environmental presence and pathogenic evolution of bacterial pathogens, such as *Campylobacter, Aliarcobacter, Salmonella, and Vibrio* (Table 1).

The Gram-negative bacterium, *Campylobacter jejuni,* is a leading cause of global bacterial foodborne gastroenteritis (244–246), with majority of cases stemming from environmental exposure to contaminated water and food (247–249). Once phagocytosed by epithelial cells lining the human intestinal tract, the *C. jejuni*-containing vacuole deviates from the canonical endocytic pathway and evades lysosomal fusion (250–257). However, *C. jejuni* does not evade lysosomal fusion in human macrophages (248, 254), indicating a cell-specific intracellular adaptation of *C. jejuni* to epithelial cells.

Since *C. jejuni* cannot grow under atmospheric conditions unless surviving in dormant biofilms (258), the bacterium relies heavily on free-living protists to support survival outside the mammalian host (249, 259–264). After phagocytosis of *C. jejuni* by amoebae, a fraction of the intracellular bacteria survives lysosomal fusion and is exocytosed back into the environment (248, 263–266). *C. jejuni* co-incubated with *Acanthamoeba* shows enhanced cell invasion and resistance of lysosomal fusion for both amoebae and human epithelial host cells compared to *in vitro* grown *C. jejuni* (263, 264). The exocytosed *C. jejuni* from *Acanthamoebae* invade nearby amoebae and evade lysosomal fusion more efficiently, compared to *in vitro* grown bacteria (248, 263). In addition, *C. jejuni* also withstands amoeba encystation during times of environmental stress (263, 267, 268). Long-term co-incubation of *C. jejuni* within *Acanthamoeba* enhances the resistance of *C. jejuni* to lysosome acidity and enhances cell-to-cell transmission (263, 269, 270). The bacterial cytolethal-distending toxin may contribute to various aspects of bacteria-host interaction (271). Co-incubation of *C. jejuni* with amoebae results in increased expression of bacterial resistance genes for nitrosative, oxidative, and other environmental stressors (262, 263, 270, 272, 273), along with increased regulation of metabolic requirements and gene expression to withstand intracellular nutrient restriction and vacuole acidity (269, 272, 274, 275). The role of various secretion systems of *C. jejuni* in the interaction with amoebae is not known but should be explored (276). Therefore, the survival of *C. jejuni* within amoebae is thought to have facilitated pre-adaptation to intracellular life and enhanced the pathogenicity of *C. jejuni* to mammalian cells (33, 260, 263, 264, 270). Taken together, the pre-adaptation of *C. jejuni* to survival in environmental amoebae continues as a training ground that has already facilitated bacterial survival in the environment and enhanced infectivity to mammalian epithelial cells.

*Aliarcobacter butzleri* is a Gram-negative bacterium belonging to the *Campylobacteraceae* family and is considered an emerging pathogen that causes severe diarrhea, enteritis, and bacteremia (277). *A. butzleri* has been isolated from multiple environmental water sources, meat, vegetables, and dairy, indicating that it can adapt to various environmental conditions similar to *C. jejuni* (278–281). Furthermore, *A. butzleri* possesses several virulence genes that are homologous to *Campylobacter jejuni* genes (282). *S*imilar to *C. jejuni*, *A. butzleri* is phagocytosed by *A. castellanii* into vacuoles where they survive for at least 10 days (283). *A. butzleri* enters *A. castellanii* through carbohydrate ligand-receptor interaction and phagocytosis is dependent on host actin polymerization (284), PI3K, RhoA, and a protein tyrosine kinase (284). Following phagocytosis, the bacterium evades amoeba predation, as transmission electron microscopy images show intact *A. butzleri* containing vacuoles that do not fuse to the lysosomes within *A. castellanii* (284). While *A. butzleri* survives within *A. castelanii*, the bacteria fail to replicate. The mechanisms of survival of *A. butzleri* within amoebae are not known, but studies have shown transcriptional changes in flagellar and putative virulence genes during intracellular survival (285). Therefore, it is likely that the *A. butzleri*-amoebae interactions have primed *A. butzleri* to infect and cause disease in mammals, and to continue to evolve and adapt to the intracellular niche within amoebae.

*Salmonella enterica* is a Gram-negative bacterium and is classified into hundreds of serovars (286). The *S*. Typhimurium serovar is transmitted to diverse hosts, including humans, through the consumption of contaminated animal-based food (287–290). Within mammalian macrophages, this pathogen-containing vacuole matures to an acidified late-endosome-like phagosome that evades lysosomal fusion, and this unique phagosome biogenesis is governed by the T3SS-2. *S*. Typhimurium can replicate within *D. discoideum* (291, 292), and known virulence genes of *Salmonella* (*aroA*, *invA, ssaD, clpV, phoPQ,* and *waaL*), as well as inorganic polyphosphate (polyP), are required for survival within *D. discoideum* (293–295). Importantly, the SopB and SifA T3SS-translocated effector proteins are required for intracellular replication of *S*. Typhimurium in *D. discoideum*, similar to mammalian macrophages (285, 296). Proteomic analyses of the *Salmonella*-containing vacuole collected from *sopB* and *sifA* mutant strains showed various proteins involved in degradation pathways, including ubiquitin ligase, COP9 signalosome, and autophagy-related proteins similar to mammalian cells (297). Therefore, it is possible that *S*. Typhimurium-amoebae interactions have shaped the pathogenic evolution of this bacterium to infect mammalian cells.

*Vibrio cholerae* is a waterborne bacterium and a causative agent of acute diarrheal (298–300). After ingestion of contaminated food or water, *V. cholerae* colonize gut epithelial cells (298, 301, 302). While *V. cholerae* is primarily encountered in brackish coastal waters and rarely contracted in industrialized nations (303, 304), recent outbreaks of severe cholera are suspected to be related to increasingly warmer climates (305) and the association of *V. cholerae* with free-living aquatic amoebae (299, 306–311). *Vibrio* can be within biofilm microbial communities, which enhances its environmental fitness (312). Predation by environmental amoebae applies selective pressure on *V. cholerae*, promoting both survival and virulence of the pathogen that carries over to the human host (306, 308, 313, 314). After phagocytosis by *A. castellanni*, *V. cholerae* is trafficked through at least two intracellular routes (308, 315). First, *V. cholerae* trapped in small or large food vacuoles may follow the canonical phagolysosomal pathway, survive digestion, and be exocytosed from the amoebae back into the environment with enhanced resistance to acidic environments (308, 315, 316). The second route, *V. cholerae*-containing vacuoles may fuse with the amoebae contractile vacuole, an osmoregulatory organelle, and replicate within the vacuole until host cell-lysis releases an abundance of bacteria back into the environment (308, 315–317). The HapA zinc metalloprotease of *V. cholerae* protects amoebae contractile vacuole from premature lysis during active colonization and intra-vacuolar replication of *V. cholerae,* indicating a pathogenic ability for promoting long-term equilibrium between *V. cholerae* and predatory amoebae (308, 315).

Intriguingly, the long-term interaction of *V. cholerae* with *Acanthamoebae* positively selects pathogenic traits with enhanced survival in amoebae, which may enhance the ability of *V. cholerae* to colonize mammals. Global transcriptomic analyses of *V. cholerae* co-cultured with *Acanthamoeba* for up to 90 days showed significant increases in gene expression associated with survival competitive fitness within amoebae and enhanced protease activity by toxins like HapA (318). Therefore, the enhanced virulence of amoebae-associated *V. cholerae* may illustrate how the co-evolution of *V. cholerae* with amoebae as an environmental host drives evolution and pathogenic adaptation to infect eukaryotic cells.

## MODULATION OF PATHOGENESIS OF *PSEUDOMONAS AERUGINOSA* BY AMOEBAE

*Pseudomonas aeruginosa* is a ubiquitous Gram-negative, opportunistic pathogen that causes pneumonia, folliculitis, osteomyelitis, keratitis, and many other diseases in both immunocompromised and immunocompetent human hosts (319–326). *P. aeruginosa* is commonly found in water systems where it thrives by forming biofilms (327–329) and interacting with amoebae, which play an important role as a reservoir of *P. aeruginosa* (321, 327, 328, 330–332). The coexistence with protozoa in biofilms enhances defensive and exploitative traits of *P. aeruginosa*, resulting in enhanced pathogenicity toward mammalian hosts (327, 333–335). Co-culture of *P. aeruginosa* with *Acanthamoeba castellanii* for 42 days significantly increases gene expression of numerous *P. aeruginosa* genes, including the T3SS (327, 336–339), which correlates with rapid death of amoebae harboring the bacteria, along with a decrease in degradation of internalized *P. aeruginosa* by neighboring amoebae (327). This bacteria-amoeba association has clearly played a major role in bacterial ecology and its aquatic presence, which is the major source for transmission to humans. Through co-evolution with amoebae, *P. aeruginosa* takes on a phenotype for evading amoebae predation and enhancing long-term survival and antimicrobial resistance that carries over to the human host (340).

Genetic analyses of *P. aeruginosa* co-cultured with *A. castellanii* have revealed a significant decrease in virulence phenotypes related to bacterial motility, pyoverdine production, and rhamnolipid production (334, 341–343). This is similar to what clinical studies show in *P. aeruginosa* collected from cystic fibrosis patients (334, 341–343). Although the specific mechanisms behind the decrease in *P. aeruginosa* virulence phenotypes are still unknown, selection and co-evolution of *P. aeruginosa* with amoebae may enhance the adaptation of *P. aeruginosa* for a commensal-dominant and chronic lifestyle seen in cystic fibrosis patients (334, 344).

## ASSOCIATION OF FUNGI WITH AMOEBAE AND ITS ROLE IN PATHOGENESIS

Many pathogenic fungi are free-living saprophytes throughout the soil that lack host specificity and cause disease in many mammalian hosts. Due to their promiscuous nature, it is not surprising to discover that interactions between environmental fungi and phagocytic amoebae have shaped the evolutionary selection for virulence traits in many fungal pathogens (345). Early studies exploring the co-culture of *Torula famata*, *Candida albicans,* and other dimorphic fungi with *A. castellanii* revealed induction of filamentous fungal forms and positive selection for growth of hyphal fungal cells resistant to protozoa predation (8, 33, 346, 347). For the fungal pathogen *Cryptococcus neoformans*, interactions with free-living amoebae have been documented for nearly 100 years (348).

Among all fungal species currently known to associate with an amoeba host, *Cryptococcus sp*. is the most extensively studied (Table 1). Surrounded by a polysaccharide capsule, *C. neoformans* and *C. gattii* resist predation by both amoebae and mammalian cells (8, 349, 350). Interestingly, exposure of *C. neoformans* to phospholipids secreted by *A. castellanii* and macrophages during phagocytosis promotes a protective stress response of the fungi, resulting in an increased capsule size (8, 33, 351–353). In addition, the co-culture of *C. neoformans* with *Dictyostelium discoideum* promotes rapid capsule enlargement and melanin production (melanization), which protects *C. neoformans* from free radicals and microbicidal peptides (351, 353, 354). Increased capsule size and upregulated melanization for *C. neoformans* and *C. gattii* following amoeba infection have been found to disrupt various innate immune processes in both *in vivo* models and *in vitro* mammalian cell cultures (8, 350, 353–355). This includes inhibiting deposition of complement on fungal cell surface, preventing antigen presentation, impeding macrophage phagocytosis, disrupting inflammatory cytokine production, and enhancing fungal resistance to free radicals and antifungal drugs (8, 350, 353–355).

Decreased expression of the *C. neoformans* transcription factor Bzp4 gene was found to be associated with reduced melanization and increased susceptibility of *C. neoformans* to amoeba predation (356). However, despite the joint effects of *BZP4* on melanin production by *C. neoformans* and resisting amoeba predation, there is no known relationship between the *BZP4-specific* genotype and *C. neoformans* virulence during *in vivo* studies (356).

Amoebae infection by *C. neoformans* also upregulates the production of additional fungal virulence factors found to have different pathogenic roles in mammalian cells (351, 357). When co-cultured with *D. discoideum*, *C. neoformans* exhibits an increased production of urease that enhances nutrient acquisition and formation of fungal extracellular vesicles (EVs) involved in disruption of amoebae metabolism (33, 351, 358). In addition to its role in amoeba, urease has been shown to promote invasion of *C. neoformans* through the blood-brain barrier of *in vivo* models, while fungal EVs inhibited macrophage response to infection (351, 357).

Other less studied fungi such as *Histoplasma capsulatum, Aspergillus sp., Candida sp.,* and *Fusarium sp*. are also reported to associate with environmental amoebae and acquire enhanced virulence (Table 1) (351, 359). *H. capsulatum* and *Fusarium sp*. exhibited enhanced virulence when co-cultured with *A. castellanii* (8, 359, 360), while co-culture of *Aspergillus fumigatus* with *A. castellanii* resulted in intracellular germination by cell metabolites and subsequent amoebae death from cell permeabilization, similar to what is observed in macrophages (351). Interestingly, the co-culture of *A. fumigatu* with *D. discoideum* or *Entamoeba histolytica* enhanced several fungal virulence factors found to play a significant role in the disease of mammalian models (351). Fumagillin significantly inhibits the growth of co-cultured *E. histolytica* and promotes enhanced epithelial cell damage during fungal invasion of mammalian cells (351, 361), while DHN melanin inhibits phagocytosis of *A. fumigatus* by amoeba and interfered with lysosomal acidification in mammalian macrophages (351, 362, 363).

While *Candida sp.-*amoebae interactions are extremely understudied, internalization of yeast cells by *V. vermiformis* was shown to enhance their survival and proliferation in tap water (351). It is not yet clear whether HGT has occurred between amoebae and fungi, but it would not be surprising if future studies revealed fungi-amoeba HGT as an additional driver for the evolution of pathogenic fungal species towards mammalian hosts.

## GIANT VIRUSES OF AMOEBAE AND THEIR ROLES IN THE PATHOGENIC EVOLUTION OF INTRA-AMOEBAE MICROORGANISMS

A giant virus in *Acanthamoeba* cell cultures (*Acanthamoeba polyphaga mimivirus;* APMV; mimivirus) was discovered in 2003 and since then at least 100 new strains of mimivirus have been isolated from water, soil, insect, and human samples through culturing with *Acanthamoeba sp*. and *V. vermiformis* (Table 1) (364–367). Environmental chlamydiae harbors a larger number of protozoa-related giant virus genes compared to pathogenic chlamydiae (Table 1) (27, 32, 53, 72, 74, 79).

Aside from *M. sibericum*, giant viruses enter amoebae and macrophage hosts through phagocytosis and undergo fusion of their membrane with the host vacuole membrane to release their genomic contents into the amoebae cell cytosol (365, 368, 369). The ability of giant viruses to enter amoebae through natural phagocytosis suggests an altered external morphology that may facilitate infection of a broader host range without the need for traditional cell invasion (366, 369). While the specific replication of each giant virus has yet to be thoroughly explored within amoeba, incorporation of the viral genome into the chromosome of amoebae is the most likely cause for rapid intracellular proliferation and amoebae cell lysis, releasing viral particles into the environment (366, 370). Unlike majority of the giant viruses, *M. sibericum* particles are released into the environment by exocytosis as opposed to amoebae cell lysis (366). Intracellular replication within *A. castellanii* and *V. vermiformis* provides a niche that protects mimiviruses from harsh environmental factors such as UV radiation, temperature, and pH (8, 367, 371). Amoebae co-culture with environmental and human samples has significantly enhanced the discovery of other giant viruses, including *marseilleviruses, pandoraviruses, pithoviruses, faustoviruses*, and *Mollivirus sibericum* (365, 371–373). Patients infected with giant viruses, particularly mimivirus, were reportedly exposed to water contaminated by amoebae containing viral particles (374–376).

The most prevalent giant virus in chlamydiae is *Megavirus chiliensis*. A total of 1,338 genes of environmental chlamydiae are found in the giant virus, but only two genes are shared with pathogenic chlamydiae (27, 32, 53, 72, 74, 79). Genomic sequencing analysis of mimivirus confirmed a significant portion of genes encoding pathogenic factors have been inherited from other amoebae-infecting microorganisms such as *L. pneumophila,* or *vice versa* (8, 375). Phylogenic analysis with the most prevalent sets (*Megavirus chiliensis* and *Protochlamydia* EI2 or *Chlamydia trachomatis* L2 434Bu) showed the presence of orthologs between these organisms with several being clustered. These findings indicate the lateral gene transfer between protozoa-related giant viruses of the family *Mimiviridae* and chlamydiae, and the inter-kingdom HGT between *Chlamydia* and giant viruses of amoebae.

While studies on interactions of giant viruses are in the early stages of development, there is sufficient evidence to indicate that environmental amoebae play a key role in promoting transmission and enhanced pathogenicity of giant viruses and viral particles throughout the environment and mammalian hosts. It is also important to note that multi-directional HGT which includes giant viruses and other intra-amoebae microorganisms is an element of continuous evolution of other microorganisms within amoeba.

## CONCLUSIONS

The interaction of microbes with environmental amoebae represents a “training ground” for the evolution and adaptation of microbial pathogens. Many microbial species have evolved to evade degradation by predatory amoebae and many of them have been equipped with the tools to replicate within this predatory environmental unicellular phagocytic organism. Through multi-directional inter- and intra-kingdom HGT, the intracellular selection pressure and subsequent adaptation within diverse amoeba hosts harboring intracellular microbial residents have shaped the pathogenic evolution of various microbes. Amoebae and macrophages are biologically similar in terms of phagocytic functions, vesicle traffic, and various nuclear functions. Therefore, evolutionary adaptations to exploit highly conserved eukaryotic processes and functions have facilitated the expansion of the host range to mammals. There is a correlation between the number of eukaryotic-like domains encoded in a bacterial genome and the lifestyle of environmental bacteria. Bacteria living in complex interactions with biofilms communities and among grazing protozoa, such as *Legionella* spp. and environmental Chlamydiae, display an enrichment in eukaryotic-like domains. The vast number of eukaryotic-like proteins and protein domains encoded by the *Legionella* and environmental Chlamydiae genomes are tandem repeats-containing proteins involved in protein-protein and protein-chromatin interactions and in modulation of host chromatin and ubiquitin-related processes, which are highly conserved in eukaryotes, including mammals. This suggests that these domains are particularly important in the interference of the pathogens with these host pathways to facilitate survival and replication in a eukaryotic cell.

Continued research efforts to decipher the role of protozoa in the evolution of pathogenic microbes should stimulate future comprehensive molecular, cellular, and genomic studies on additional protozoan species. Investigations into the control of pathogenic microbes in the environment, such as water resources, should take into account the intra-amoebae presence of pathogenic microbes and their protection within amoebae, particularly the cyst form, from harsh environmental conditions and standard decontamination strategies (377). In addition, intra-amoebae microbes and their dormancy are likely to be more resistant to standard antibiotics, and studies should be performed to evaluate this possibility (378, 379). Moreover, it may not be surprising that the infectious particles in mammals contain an amoebae harboring infectious microbes. This would be as a Trojan horse of pathogenic microbes that are infectious and are protected from the innate immune response of mammals. This possibility would impact studies on the infectious dose for mammals as well as the virulence capacity of the infectious microbe within amoebae. These major gaps in our knowledge should be pursued for future studies.

Despite the major progress made in deciphering the wide variety of microbe-amoebae interactions, additional mechanistic studies are needed to uncover their true and vast complexities along with their major roles in the pathogenic evolution of various microbes. More diverse species of amoebae, besides *Acanthamoeba* and *Dictyostelium* species, need to be included in studies with microbial pathogens. However, the continued paucity of various research tools along with minimal genomic and metagenomic studies on amoeba species will continue to hamper our progress. These limitations must be overcome to expand our knowledge of how pathogenic microbes have co-evolved and adapted to the intra-amoebae environment, and the role of this co-evolution in the infection of mammals. Although our current understanding has improved, it remains in the infancy of this remarkable genetic melting pot of multi-directional HGT and its role in the evolution of microbes capable of causing various infections in mammals. Our current knowledge remains the tip of the iceberg but it is hopeful that with rapid technological advances and reduced cost of genomic analyses, additional studies will likely reveal novel manipulations of eukaryotic processes of unicellular amoebae hosts by microbes. Importantly, studies will continue to unravel the evolutionary biology of eukaryotic processes in unicellular eukaryotes and their continued evolution in multi-cellular eukaryotes, and how amoeba-adapted microbes have evolved and expanded their host range to infect evolutionarily distant hosts. Amoebae have played, and will definitely continue to play, major roles as a training ground for the evolution of microbial pathogens that infect mammalian hosts and other multi-cellular eukaryotes.
